# Quantum Dot Solar Cells: Background, Progress, and Perspective

**DOI:** 10.3390/mi17040474

**Published:** 2026-04-15

**Authors:** Kumar Neupane, Jeff Kabel, Join Uddin, Raksha Dubey, Rojina Ojha, Dongyan Zhang, Yoke Khin Yap

**Affiliations:** 1 Department of Physics, Michigan Technological University, 1400 Townsend Drive, Houghton, MI 49931, USA; kumarn@mtu.edu (K.N.); jskabel@mtu.edu (J.K.); juddin@mtu.edu (J.U.); fraksha@mtu.edu (R.D.); rojha2@mtu.edu (R.O.); dozhang@mtu.edu (D.Z.); 2 Elizabeth and Richard Henes Center for Quantum Phenomena, Michigan Technological University, 1400 Townsend Drive, Houghton, MI 49931, USA

**Keywords:** quantum dots, solar cells, fourth-generation solar cells, perovskite, carbon quantum dots

## Abstract

The discovery of quantum dots (QDs) earned a Nobel Prize and has led to widespread applications in research and technology. In this review, we focus on the use of QDs in solid-state solar cells (QDSCs). We begin with an overview of the basic principles of SCs. Then, we discuss how device architecture has developed over recent decades, setting the stage for the final section on fourth-generation solar cells (Perspective section). We also highlight progress in material development, starting with lead- and cadmium-based QDs and progressing to more recent carbon- and perovskite-based QDs. Additionally, we review materials used for electron-transport layers (ETLs) and hole-transport layers (HTLs). The articles also present recent advances in QDSCs across various QD types. In the final section, we recommend that future research focus on three main areas: QD active-layer materials, material interfaces, and device architecture. These efforts could lead to sustainable QDSCs that potentially surpass the Shockley–Queisser (SQ) limit.

## 1. Introduction

The rise of artificial intelligence (AI) is driving a drastic increase in electricity use. Renewable energy from photovoltaic (PV) devices is a sustainable solution that can meet growing energy demands [1]. Solar cells (SCs) based on crystalline silicon wafers offer the best photoconversion efficiency of around 27.03% [2], approaching the theoretical limit of 29.4% given by the Shockley–Queisser (SQ) limit for single-junction cells [3]. Such SCs are exceptional but not without shortcomings. Single-junction SCs absorb only a limited range of the solar spectrum, determined by silicon’s band gap. Other limitations in single-junction SCs include the cooling of photoexcited carriers to the lattice (thermalization) [4], transmission loss of unabsorbed photons, and thermodynamic losses to the environment [5].

**Figure 1:**
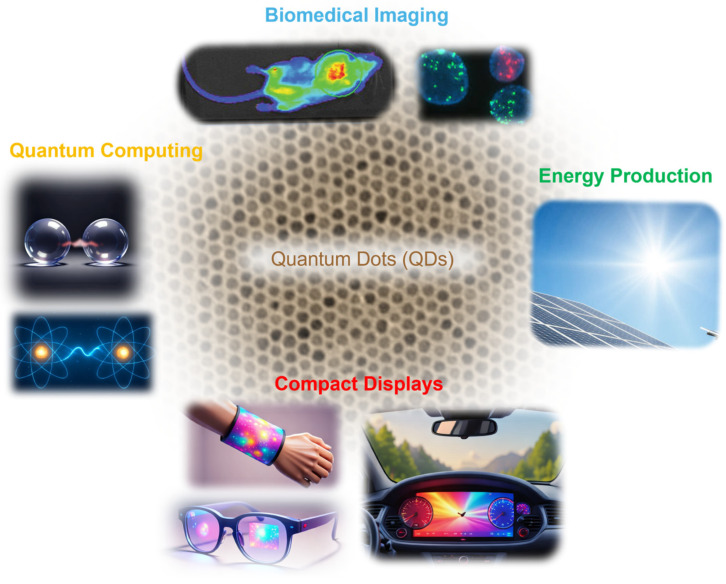
Schematic representation of QDs’ applications. Graphics are generated by *Image Playground* from Apple and *Gemini* from Google.

On the other hand, quantum dots (QDs) with widely tunable band gaps would likely lead to multijunction SCs that would overcome the SQ limit. The pioneering work on semiconducting QDs in the 1980s led to a Nobel Prize in Chemistry 2023 [6,7,8]. Since the band structures, energy band gaps, and optical properties of QDs can be engineered by composition and diameter, research on these nanocrystals has expanded across various aspects over the past three decades. In terms of material type, scientists have developed various cadmium (Cd)- and lead (Pb)-based chalcogenide QDs, including CdS and CdSe, which emit visible light, and PbS and PbSe, which emit near-IR light. There have been many efforts to create Cd- and Pb-free QDs. Some examples include indium (In)- and zinc (Zn)-based QDs, such as InS, InSe, InP, InAs, ZnS, and ZnSe. The band gaps and optical properties of these binary QDs can further be modified into ternary alloyed QDs, such as CuInS_2_ (CIS), AgInS_2_ (AIS), and InGaAs, to name a few. More recently, carbon-based and perovskite QDs have attracted significant attention. In terms of applications, QDs have been used for bioimaging, solar cell energy production, compact displays via light-emitting diodes (LEDs), and quantum computing. The QD application landscape is illustrated in Figure 1.

Given the tunable band gap in QDs, their use in SCs enables us to capture a wider range of the solar spectrum, thereby enhancing power output beyond the SQ limit for single-junction cells. Therefore, in this review, we focus on examining the photovoltaic applications of QDs to point the way for future photovoltaic research. Specifically, we review quantum dot solar cells (QDSCs) and not QD-sensitized SCs (QDSSCs). For context, QDSCs are solid-state devices. In contrast, QDSSCs consist of a mixed powder/paste of QDs and semiconductors (such as TiO_2_ and ZnO), and typically involve redox processes. We will first provide a brief overview of the basic principles of SCs in Section 2. In Section 3, we will review the evolution of QDSC device architecture. In Section 4, we will discuss the materials aspects of QDs, electron-transport layers, and hole-transport layers. In Section 5, we will summarize some examples of photovoltaic devices by the type of active materials (photon-absorbing QDs), i.e., those using Pb- and Cd-based QDs, as well as those with carbon and perovskite QDs. Finally, in Section 6, we will present our perspective on future research and point the way towards fourth-generation QDSCs.

## 2. Basic Principles of Quantum Dot Solar Cells (QDSCs)

In solar cells, the band gap of semiconducting photon-absorbing material (also known as the active layer, AL) determines the range of the solar spectrum that can be absorbed and converted into useful electrical power. Lower-energy photons, those with energies below the AL band gap, will not be absorbed; similarly, higher-energy photons, those with energies above the band gap, lose excess energy via thermal loss. The key advantage of using QDs in SCs is their tunable bandgap, which enables scientists to maximize light absorption across a wider spectral range.

First, let us examine the quantum confinement effect, which is responsible for the tunable band gaps in QDs as a function of their diameter. The confinement effects in QDs are related to the exciton Bohr radius, which refers to the physical separation between an electron in the conduction band and the associated hole in the valence band. As the radius of each semiconductor material decreases, there are fewer atoms and electrons within the QDs. This leads to fewer energy states within the energy bands. A reduction in the energy-state density within the energy bands increases the energy band gap [9]. For example, lead sulfide has an exciton Bohr radius of 18 nm, meaning that below 18 nm, quantum effects are significant. In contrast, TiO_2_ has a Bohr radius of about 1.5 nm, so the quantum confinement effect is unlikely to be observed in nanoparticles, as their diameters are larger than the Bohr radius [10].

The effective band gap of a QD, due to the quantum confinement effect, can be expressed by the Brus equation [11] as:

(1) $$\Delta E\left(r\right)=E_{gap}+\frac{h^{2}}{8r^{2}}\left(\frac{1}{m_{e}^{*}}+\frac{1}{m_{h}^{*}}\right)-\frac{1.786{\;e}^{2}}{4\pi \varepsilon_{o}\varepsilon_{r}r}$$

where *r* is the radius of the QD, *h* is Planck’s constant, and $m_{e}^{*}$ and $m_{h}^{*}$ is the effective mass of the electron and the hole, respectively. The second term represents the confinement energy [12], and the third term is the Coulomb term for electron–hole attraction. This relationship enables precise tuning of the absorption onset and energy levels through synthetic control of QD size, thus leading to various optoelectronic properties based on their size [13].

Quantum dot solar cells (QDSCs) are PV devices that use semiconducting QDs as the AL [14]. Unlike quantum dot-sensitized solar cells (QDSSCs), which use QDs as sensitizers in liquid electrolyte systems, QDSCs are solid-state devices that form conventional p-n junctions, Schottky junctions, or heterojunctions using QD films as the active semiconductor layer [15,16]. QDSCs have the potential to achieve thermodynamic conversion efficiencies up to 66% by utilizing hot photogenerated carriers and multiple exciton generation phenomena [17]. It is believed that QDSCs offer low costs for fabrication and reduced complexity as QDs are synthesized via wet chemistry in solution, and they can be easily deposited over a substrate via various simple, scalable, low-cost, and low-temperature solution-based techniques, such as spin-coating, spray-coating, blade-coating, or roll-to-roll printing [18,19]. The basic operation of QDSC requires the following sequence of carrier dynamics [20].

1. Active layer (AL) or photon-absorbing materials: A photon with energy higher than the optical band gap will be absorbed to generate an exciton (electron–hole pair)
2. Charge separation: The electron and hole must be separated and flow in opposite directions to complete the circuit and generate electricity. This is achieved through the electron-transport layer (ETL) and hole-transport layer (HTL), which laminate the AL at opposite ends.

### 2.1. Current–Voltage (IV) Characteristics

Solar cells operate by establishing an electric potential difference between the two electrodes. The potential difference creates a diode-like electrical device. The current–voltage (I–V) characteristics of such junctions follow the generalized diode equation, in which current flow in one direction is constant with voltage. In contrast, the current flows in the opposite direction and increases exponentially with the applied voltage. Hence, the dark current (or current at no illumination) density (per unit device area) as a function of applied voltage can be written as:

(2) $$J_{\mathrm{dark}}\left(V\right)=J_{0}\left(\exp\left(\frac{qV}{nkT}\right)-1\right)$$

where $J_{0}$ is the saturation current density, *n* is the ideality factor, which is between 1 and 2 and increases as current decreases, *q* is the electronic charge, *k* is Boltzmann’s constant, and *T* is the temperature (K) [21]. However, deviations from ideal diode behavior are expected in QDSCs due to the complex multijunction environment and transport limitations in QD systems.

Under illumination, light absorption generates additional charge carriers, increasing current flow. They must be added to the initial dark current to get the total current in the system. For a light comprised of multiple wavelengths, the total photocurrent can be calculated by summing the contributions from each wavelength. Hence, short circuit photocurrent density (*J_sc_*) is:

(3) $$J_{sc}=q\int I_{s}\left(E\right)\;QY\left(E\right)\;dE$$

where $I_{s}\left(E\right)$ represents the solar photon flux, *E* is the photon’s energy, and *QY* is the quantum yield, which gives the number of collected electrons per incident photon. Then, the net current density can be written as,

(4) $$J\left(V\right)=J_{sc}-J_{\mathrm{dark}}\left(V\right)=J_{sc}-J_{0}\left(\exp\left(\frac{qV}{nkT}\right)-1\right)$$

**Figure 2:**
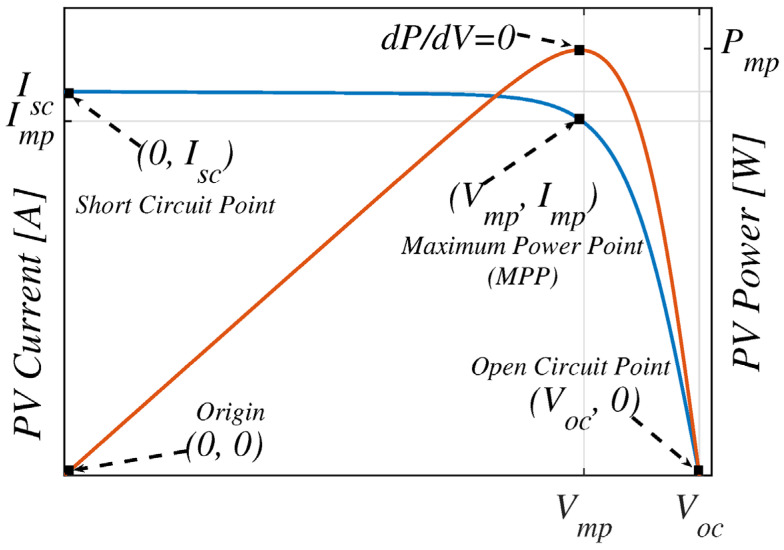
Typical PV characteristic curves, as specified in the manufacturer’s datasheet at standard test conditions (STCs). Reprinted with permission from ref [22]. Copyright 2024 MDPI.

Current (*I*) and voltage (*V*) are the measurable parameters from a solar cell test. The typical IV characteristics (blue curve in Figure 2) of QDSCs can be plotted together with the power (*VI*) curve (red).

### 2.2. Photovoltaic Parameters

#### 2.2.1. Fill Factor (FF)

The fill factor quantifies the “squareness” of the IV curve and represents the ratio of maximum power output to the product of *V_oc_* and *I_sc_*:

(5) $$FF=\frac{P_{mp}}{V_{oc}\times I_{sc}}=\;\frac{V_{mp}\times I_{mp}}{V_{oc}\times I_{sc}}$$

where *I_mp_* and *V_mp_* are the current and voltage at the maximum power point. The fill factor is primarily limited by the device’s series resistance (*R_s_*) and shunt resistance (*R_sh_*). Series Resistance is due to the resistance at the contact interfaces and transparent electrodes, etc. Higher series resistance results in a low FF and overall efficiency, particularly at high current densities [23]. Shunt Resistance defines leakage pathways in the devices. Low *R_sh_* means strong leakage, so a higher *R_sh_* is desirable. Interface defects, inadequate junction formation, etc., create shunt resistance [24].

The fill factor (*FF*) measures how close a solar cell operates to its maximum power efficiency and is defined as the ratio of its maximum power output (P_max_) to the product of its open-circuit voltage (*V_oc_*) and short-circuit current (*I_sc_*) [25]. Although FF up to 86.59% has been achieved for Si SCs, it is lower than the theoretical value of 89.26% due to the constraints of the Shockley–Read–Hall (SRH) recombination and series resistance [26]. Würfel et al. reported that in organic solar cells, the fill factor is primarily limited by recombination processes that scale linearly with light intensity, which are typically attributed to geminate or trap-mediated losses [27].

Choi et al. found that in PbSe/PbS core/shell QD heterojunction solar cells, increasing the thickness of the PbS shell increases the FF from 30% to 49% and the *J_sc_* from 6.4 to 11.8 mA cm^−2^, while maintaining *V_oc_* at 0.46 V [28]. Rosiles-Perez et al. [29] found that using 1,2,3-propanetriol increased short-circuit current density from 0.71 to 15.98 mA/cm^2^, and reached 21 mA/cm^2^ after thermal treatment of Bi_2_S_3_ QDs in a non-toxic Bi_2_S_3_ QDSSCs. The significant *J_sc_* enhancement is due to improved crystallinity of Bi_2_S_3_ QDs and better alignment of their energy levels, resulting in more efficient photogeneration.

#### 2.2.2. Power Conversion Efficiency (PCE)

The overall power conversion efficiency represents the fraction of incident solar power converted to electrical power:

(6) $$\mathrm{PCE}=\frac{V_{oc}\times I_{sc}\times FF}{P_{in}}\;$$

where *P_in_* is the incident light power density under standard illuminations (AM1.5G, 100 mW/cm^2^, 25 °C). The PCE can be improved by maximizing *I_sc_* and *V_oc_*, as discussed in the following subsection.

#### 2.2.3. Short-Circuit Current (I_sc_)

The short-circuit current is a maximum current extracted at zero voltage from the solar cell (when the cell is short-circuited), which is directly proportional to the rate of photogenerated charge collection [30]. In QDSCs, *I_sc_* is determined by several key factors, some of which are discussed here. Optical Absorption: Even if the absorption coefficient of QDs is high (10^4^–10^5^ cm^−1^), the active QDs layer thickness must be optimized to absorb solar photons efficiently to balance absorption with charge transport capabilities [31]. Quantum Efficiency: The cell’s efficiency depends on the number of collectible charge carriers generated per absorbed photon. This includes the efficiency of exciton separation and the collection probability of separated charges before recombination [32]. Transport Properties: The ability of charge carriers to reach the front and back electrodes depends on mobility and diffusion length within the QD film, which together determine the collection efficiency and thus the short-circuit current density (*J_sc_*).

Short circuit current density reflects not only the ability of QDs to absorb light, but also the efficiency with which charges are separated and transferred without recombining.

#### 2.2.4. Open-Circuit Voltage (*V_oc_*)

The open-circuit voltage (*V_oc_*) represents the maximum photovoltage that a solar cell can generate at zero current and is caused by the separation of the electron and hole quasi-Fermi levels within the absorber material [33]. Dambhare et al. reported that using cadmium halide-based solution–phase ligand exchange, a record *V_oc_* of 0.7 V was achieved in PbS QD solar cells, attributed to enhanced surface passivation and decreased sub-bandgap trap states [34].

The open-circuit voltage is the maximum photovoltage achievable under zero-current conditions. In QDSCs, *V_oc_* is influenced by several factors, as follows. Band Alignment: The difference in the quasi-Fermi levels between the ETL and HTL in the QD layer determines the Voc. QDs with a larger band gap offer a high *V_oc_* value, but lose infrared absorption [35]. Recombination Losses: Non-radiative recombination is the primary factor for reducing open-circuit voltage in QDSCs. Losses come from recombination at the QD interfaces, while traps inside the QD film also contribute to the mechanisms [36]. Interface Quality: Good contact interfaces reduce series resistance and recombination rates. High-quality interfaces with minimal defects are expected to yield high *V_oc_* [37].

## 3. Progress in Solar Cell Architecture

The developments in photovoltaic devices can be classified into multiple “generations”. First-generation (conventional) photovoltaics are crystalline technologies (typically based on Si or GaAs), with planar structures consisting of a single p–n junction with front and back contacts. Second-generation (thin-film) devices are based on thin films intended to be lower-cost and flexible. Third-generation (emerging) solar cells encompass a wide range of technologies that use novel materials or architectures to surpass the Shockley–Queisser limit. Fourth-generation (hybrid/advanced nanotechnology) devices are yet to be defined, likely integrating features from previous generations while maintaining high conversion efficiency, lower cost, flexibility, and durability.

It should be noted that the term “generation” may be misleading—first-generation devices are far from obsolete, and 90% of commercial solar cells are from the first generation [38]. Our discussion will primarily focus on third-generation devices, with some speculation on fourth-generation designs. To better understand the trends in photovoltaic innovation, we will begin with a brief examination of earlier generations.

### 3.1. First- and Second-Generation Solar Cells

As previously mentioned, first-generation photovoltaics are single-junction devices, which are the most mature and dominant technology to date [39,40,41]. While these devices have seen a remarkable increase in power conversion efficiency since their conception in the 1950s, the devices suffer from high production costs and are limited to a rigid form (not flexible). To address these limitations, researchers developed second-generation thin-film solar devices.

Thin-film devices use less material than their first-generation counterparts. Examples of thin-film solar cells include those based on amorphous Si [42] and copper indium gallium selenide (CIGS) [43] films. They are referred to as second-generation solar cells [44,45]. However, the efficiency of thin-film solar cells has fallen well behind single-junction silicon and gallium arsenide devices. In the early 2000s, the best-performing thin-film devices had PCEs around 19%, while traditional silicon and gallium arsenide devices were at around 25% [46]. At the time of writing, the most efficient traditional thin-film devices have PCEs of 23.6%, while the single-junction Si and GaAs devices have PCEs of 27.8% and 29.1%, respectively [47]. The quest for lightweight, flexible, efficient photovoltaics required new materials and techniques beyond traditional thin-film technologies, and so the third generation of devices was born.

### 3.2. Third-Generation (Recent) Solar Cells

Third-generation solar cells are adopting emerging technologies, including dye sensitized solar cells (DSCs), organic/polymer solar cells (OSCs), perovskite solar cells (PSCs), and QD-based solar cells, commonly referred to as emerging solar cells, are still in the scientific research/development stage [48,49]. Some innovations in third-generation photovoltaic device architecture challenge the assumptions underlying the SQ limit, particularly with multijunction designs. The original calculations assumed a single p–n junction, and that incident light had the same luminous flux as sunlight on Earth’s surface. The earliest architectural innovations were additional p–n junctions and solar concentration. Another assumption made by Shockley and Queisser was that one photon can produce only one exciton. Additionally, the calculation indicates that a major loss mechanism is due to high-energy photons. Incoming photons with energy above the material’s bandgap can generate excitons and convert excess energy into heat within the crystal, potentially affecting the device’s long-term performance and stability. In recent years, multiple exciton generation has been observed in QDs, and devices have been fabricated using them to capture “hot” electrons with energies above the crystal’s bandgap.

#### 3.2.1. Quantum Dot-Based Solar Cell Technology

Here, we will narrow our scope to focus on QD-based technologies. QD-based solar cells are attractive because of their size- or composition-dependent tunable band gap, sufficient photostability, high extinction coefficient, strong inherent dipole moments, and overall stability. In addition to these, they have other unique characteristics, such as multiple exciton generation (MEG), hot carrier extraction potential, intermediate band gap formation, etc., which could enable overcoming the Shockley–Queisser efficiency limit for a single-junction solar cell [50]. These properties give a room for modification of absorption and energy level, potentially helping in surpassing the traditional Si-based solar cell and utilizing them for cost-effective and flexible photovoltaic applications [51].

The historical evolution of some QDSC architecture is discussed in the following subsections. The early QDSCs can be broadly divided into three phases: (1) quantum-well/p–i–n junction devices; (2) mesoscopic scaffold designs; and (3) solution-processed heterojunction devices. Each of these phases differs in both fabrication methodology and charge generation/separation. In addition, QDSCs can also be categorized by the type of interfaces/junctions (Section 3.2.2).

##### Epitaxial p–i–n Junctions with QDs

Early efforts to integrate QDs into photovoltaic devices predate the availability of colloidal nanocrystals and consequently relied on epitaxial semiconductor technologies. These first QDSC architectures aimed to enhance photocurrent by embedding QDs within the intrinsic region of p–i–n junctions, thereby enabling sub-bandgap absorption. In these devices, the intrinsic layer is not a single material layer but rather consists of multiple thin III–V semiconductor layers forming quantum well structures, stacked between barrier layers. These layers are very thin (<50 nm) and confine electrons within. These quantum wells serve as the device’s active layers, and the electric field in the intrinsic region separates electron–hole pairs, generating a measurable photocurrent. As an additional benefit, the vertical alignment and electronic coupling of QDs can be enhanced by the strain field effects of the buried QD layer [52].

**Figure 3:**
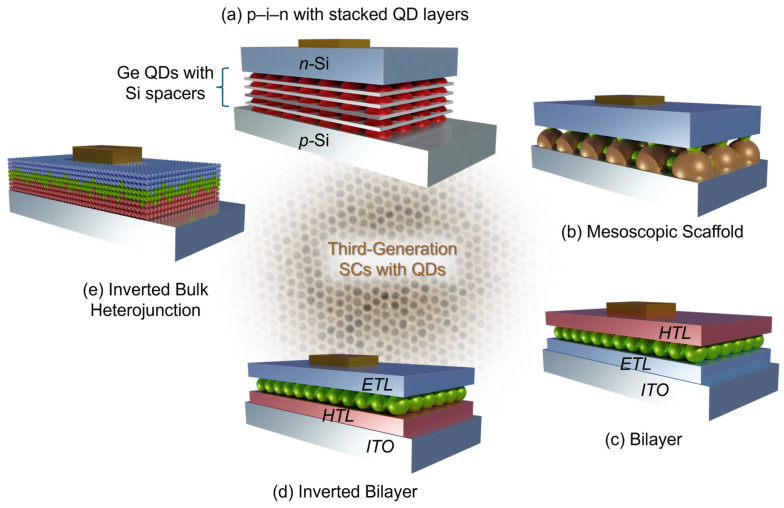
Schematic representation of third-generation QDSCs.

In their 2003 work, Alguono et al. grew Ge dots stacked in multilayer structures via the Stranski–Krastanov method [52]. The Ge dot layers (red spheres) were separated by a 39 nm-thick Si spacer layer (grey films) for a total of 50–100 layers before being topped with a 600 nm Si layer, as illustrated in Figure 3a. The p–n junction was then formed through thermal diffusion of P from a spin-coated Ohka coat diffusion source, and the Al and Ag were evaporated for the back contact and front fingers, respectively.

Many early p–i–n devices relied on the Stranski–Krastanov (SK) method, which had some fundamental limitations. The SK method yields QD arrays with low areal density, resulting in weak absorption in sub-bandgap regions [53]. Attempts have been made to circumvent the low areal density by increasing the number of QD layers, but each additional layer increases elastic strain in the lattice, resulting in degraded QD structures after about 10 layers because misfit dislocations are generated due to the accumulation of internal strain beyond the critical thickness [54,55,56], in which the compressive strain due to the QD layer was compensated by introducing a spacer layer to cause a tensile strain [57], and by doing so, they were able to fabricate devices using InAs/GaAs with 20 stacks of InAs QDs, which had improved size uniformity with a redshifted absorption edge [54]. Beyond the issues of low areal density and lattice strain effects, devices based on epitaxial QDs also suffer from the QDs’ localized electronic states, which act as unintentional recombination sites [53]. The limited tunability of epitaxial QD size and bandgap increases the difficulty of avoiding these recombination sites, and so, these parasitic exciton pathways are nearly inevitable.

##### Solution-Processed Mesoscopic Scaffolds with QDs

From a manufacturing standpoint, reliance on molecular beam epitaxy (MBE) or metal–organic chemical vapor deposition (MOCVD) to produce epitaxial quantum-well structures is undesirable due to the high cost. Ultimately, this led researchers to explore more easily scalable solution-processed methods, such as the mesoscopic scaffold architecture.

Mesoscopic scaffold devices are inspired by the architecture of dye-sensitized solar cells, in which semiconducting particles with high surface-area structures (typically TiO_2_ or ZnO particle films) serve both as scaffolds for dye molecules and as electron-transport layers. The large interfacial area between the scaffold/electron-transport layer and the dye molecules/QDs provides ample room for exciton (electron–hole pair) separation. For mesoscopic scaffold QD devices, QDs were deposited onto the scaffold, using methods such as chemical bath deposition, electrochemical deposition, and successive ionic layer adsorption and reaction (SILAR) techniques [58]. The operational principle is as follows: photoexcitation occurs within the QDs, and electrons are injected into the oxide scaffold at ultrafast rates (~10^11^ s^−1^) [59], while holes are injected into an electrolyte or other conventional transport material.

Robel et al. [59] were among the first to attempt to fabricate such a device. By injecting CdSe QDs into TiO_2_ nanocrystals, they achieved a device with a photon-to-charge-carrier generation efficiency of 12%. Figure 3b shows a schematic demonstrating how the TiO_2_ (brown spheres) particles are used as a scaffold to hold the CdSe QDs (green spheres). Over the years, others have improved device design by modifying the scaffold or the QDs. Liu et al. embedded gold nanoparticles into a TiO_2_ nanoparticle film before depositing CdSe QDs via chemical bath deposition—this led to a 50% increase in photocurrent compared to CdSe QDs on a bare TiO_2_ nanoparticle film [60]. In another study, TiO_2_ beads were solvothermal processed with ammonia to increase their porosity, and the authors found that this procedure provided high QD loading and strong light scattering, both of which contributed to improved device performance [61]. In addition to modifying the scaffold, researchers have found success by using multiple QD varieties within a single device to increase photoconversion efficiency and charge transport [58].

While mesoscopic scaffolds demonstrated that colloidal QDs could be integrated into functional photovoltaics, ultimately, they were severely constrained by physical limitations. The interface between the scaffold and QDs had high trap densities, which resulted in reduced mobility and recombination [62]. The liquid electrolytes commonly used as the hole-transport layer in early devices exhibited poor stability, which could lead to QD dissolution [62], and the solid-state replacements suffered from poor interfacial contact. Fundamentally, charge transport through these scaffold/QD networks relied on hopping or diffusion (rather than band-like conduction in newer devices), resulting in lower achievable current densities and fill factors. These limitations led to the innovation of planar and bulk heterojunction architectures.

##### Solution-Processed Heterojunction Architectures with QDs

Heterojunction QD solar cells use QD films (typically chalcogenides, such as PbS or CdSe) as the active layer, with suitable transport layers flanking the QD film to form functional p–n junctions. Heterojunction device architectures can be broadly divided into two archetypes: (1) bilayer heterojunction, in which a QD film is sandwiched between well-defined electron- and hole-transport layers, and (2) bulk heterojunction (BHJ) blends, in which QDs are blended with another semiconductor or conducting medium.

As schematically illustrated in Figure 3c, bilayer heterojunction devices are fabricated by depositing a QD absorber film by spin coating or other layer-by-layer methods on top of an electron-transport layer (ETL) and then capping the QD film with a hole-transport layer. An example of a typical stack would be a transparent conducting oxide for an ETL (such as ZnO or TiO_2_), the QD film (e.g., PbS or CdSe), a hole-transport layer (e.g., MoO_3_ or organic materials, such as spiro–OMeTAD), and finally a metal back contact. The operational principles of bilayer devices are reminiscent of conventional thin-film photovoltaics: there is a clear separation of charge generation (QD active layer) and charge transport (dedicated ETL and HTL). The energy levels of these layered materials must be aligned sequentially for efficient exciton extraction. Improvements have been made to such a basic structure. Undesirable exciton recombination can be suppressed by introducing buffer layers. Additionally, buffer layers have been shown to enhance both the open-circuit voltage and short-circuit current. In one study, the power conversion efficiency of a device increased from 6.0% to 7.5% when introducing a buffer layer of CdSe QDs between the active layer (PbS) and the ETL [63]. Figure 3d shows the inverted bilayer structure.

Bilayer and inverted bilayer architectures face some fundamental limitations. The thickness of the QD film must be carefully controlled; if it is too thin, absorption is insufficient, and if it is too thick, charge transport suffers from reduced carrier mobility and a higher recombination probability. Colloidal QD films often require a ligand exchange to replace longer, more insulating surface ligands with shorter, more conductive species. These ligand exchanges can be tricky to control, and it is not uncommon for the process to degrade QD quality or lead to inhomogeneous coatings. As a result, bilayer device performance typically plateaus, and consequently, interest shifts towards other architectures.

To circumvent the design trade-off between light absorption and carrier transport found in bilayer architectures, researchers adapted the bulk heterojunction (BHJ) concept from organic photovoltaics. As illustrated in Figure 3e, in a BHJ architecture, QDs are blended or co-deposited with another semiconductor or conducting medium (such as a conjugated polymer) to form an interpenetrating donor–acceptor network. The interpenetrating nature of the network allows charge separation and transport throughout the material’s volume. The foundation of this work was laid by demonstrating that a mixture of n-type and p-type nanocrystals improves device performance over bilayer architectures. In their 2012 work, Rath et al. fabricated devices using ITO, PbS QDs, and Bi_2_S_3_ nanocrystals in both bilayer and BHJ architectures, and the BHJ device reached a PCE of 4.87%—a threefold improvement over the bilayer device [64]. More refined implementations of this architecture using p- and n-type QDs achieved PCEs exceeding 10%, roughly doubling the performance of bilayer counterparts [65].

In more recent years, the BHJ QD architecture has been applied in tandem with organic or perovskite components. By dissolving conjugated polymers in a perovskite–QD matrix solution, a polymer–QD BHJ hybrid layer can be created at the QD/HTL interfaces. This significantly enhances the short-circuit current density and consequently the PCE [66]. This approach reduces recombination losses and can deliver PCEs around 14% [66].

The BHJ architecture continues to be developed. It offers many advantages—a high interfacial area enables exciton dissociation, and shorter diffusion paths reduce the probability of recombination. These help to mitigate some of the limitations of QDs that plague bilayer devices, namely, low mobility and trap-mediated recombination. Nevertheless, BHJ architecture comes with its own complexities. Achieving a well-controlled morphology with continuous carrier pathways can be challenging and requires careful management of the relative domain sizes and phase segregation. These challenges increase the difficulty of maintaining high-quality, high-uniformity films, and reproducibility often suffers as a result.

#### 3.2.2. Categorized by the Junction-Type

As discussed, QDSCs can be differentiated by the stacking order of materials. These devices can also be categorized by the type of electronic junction they contain, as tabulated in Table 1.

(i) Depleted heterojunctions: These devices form a depletion region at the interface of the QDs and the metal oxide electron-transport layer (ETL) [67]. This depletion region often extends into the QD active layer near the oxide interface, enabling drift-assisted charge collection and mature oxide processing. The *V_oc_* and FF are frequently limited by band-offset and recombination at the ETL/QD interface. Such issues have motivated the strategy of interfacial passivation or buffer layer (see discussion below).
(ii) Metal/semiconductor (QDs) Schottky junctions: These devices consist of a thin p-type QD (eg, PbS, PbSe, etc.) film sandwiched between the transparent electrode with ohmic contact and the metal back electrode with low work function. Such a configuration forms a rectifying Schottky barrier, generating a built-in field. The advantages are the simplicity (lower fabrication cost), enhanced light absorption, improved carrier transport and collection, flexibility, and versatility [68]. However, it has low *V_oc_*, high interfacial recombination at the metal–QD interface, imbalance charge transport, stability issues, etc [69].
(iii) All-QD p–n junctions (or p–i–n or n–i–p): These devices are based on “quantum-junction”, which uses both the p-type and n-type colloidal QDs or modified films to form a quantum-to-quantum heterojunction. This junction is similar to a conventional p–n diode, but uses QDs to create a built-in electric field [70]. The main advantage is superior band alignment and reduced energy loss at the interface, which can boost the open-circuit voltage and efficiency [71]. However, it faces challenges, including complex processing for balanced charge transport, difficulty in controlling doping levels, and persistent charge recombination in the quasi-neutral and depletion regions [72].

### 3.3. Fourth-Generation Solar Cells: Are We There Yet?

There is no consensus on the accurate definition of fourth-generation solar cell architecture. Likely, we have already started, or we are still uncertain. Here, let us discuss some advanced QDSC designs that may point the way. Again, we are still focused on QDs. It is likely that scientists will creatively incorporate other low-dimensional materials to create fourth-generation architecture. Some of the contents to be discussed in Section 6 (Future Perspective section) may guide the development of fourth-generation SCs. Here, we would like to define fourth-generation solar cells as follows.

Fourth-generation SCs must offer PCEs that significantly surpass the single-junction Shockley–Queisser limit, while being more durable, scalable, and cost-effective than the single-crystalline Si SCs. These SCs must be compatible with humans and the environment and add new functionality, such as being flexible and wearable, without being limited by any form factors.

## 4. Materials for Quantum Dots, Electron-Transport Layer, and Hole-Transport Layer

### 4.1. Lead- and Cadmium-Based Quantum Dots

Ekimov et al. demonstrated the first experimental confirmation of quantum confinement in Cd-based semiconductor microcrystals [73]. They found that the absorption spectrum of the CdS microcrystal showed a wavelength shift that correlated with the crystal size. Following this pioneering work, Cd-based QDs made rapid progress in size-homogeneous synthesis, core–shell passivation, and ligand exchange [74,75,76]. As a result, Cd-based QDs are used in a variety of applications, including bioimaging, light-emitting diodes (LEDs), SCs, and optoelectronic devices [74,77,78,79,80,81].

Similarly, Nenadovic et al. were the first to report the synthesis of Pb-based QDs and found that, due to the quantum confinement effect, the absorption spectrum of PbS particles shifts to higher energy than that of bulk materials [82]. Significant advancements in tunable size, surface passivation, core–shell surface passivation, and ligand exchange were achieved by Pb-based QDs following the initial work [83,84,85]. Consequently, Pb-based QDs are used for LEDs, SCs, sensors, optoelectronic devices, and biomedicine [81,85,86,87,88].

QDs have attracted interest in third-generation photovoltaics research due to their affordability, simple fabrication, lightweight nature, and flexibility in QDSCs [89]. QDSCs could likely capture multiple spectral bands of sunlight more efficiently than traditional silicon cells, resulting in higher conversion efficiency [90]. Cd- and Pb-based QDs are popular due to their well-established synthesis techniques [91], which allow tunable optical and electronic properties by adjusting their compositions and sizes [89,90,92,93,94]. For instance, QDSCs based on Cd chalcogenides (CdX, X = S, Se, or Te) have received considerable attention due to their efficient light absorption across a range of band gaps: CdTe (1.49 eV), CdSe (1.73 eV), and CdS (2.25 eV) [92].

There are ongoing efforts to develop novel synthesis methods. Wang et al. reported that CdSe QDs can be synthesized within solid polyvinyl chloride (PVC) or polyvinylidene difluoride (PVDF) using a polymer-template route, in which the polymer’s halogen ligands control nanocrystal growth through surface interactions [95]. This work demonstrates the in situ growth of CdSe QDs within PVC and PVDF templates via spin coating or inkjet printing in air at mild temperatures, without the use of additional ligands, resulting in environmentally stable QDs. By coordinating with surface Cd atoms, the halogen atoms in the polymers eliminate defects, thereby enhancing the photoluminescence quantum yield and providing excellent thermal and chemical stability. Thambidurai et al. used a chemical precipitation approach to produce CdS QDs [96]. They found that the grain size is roughly 2.5 nm and that the hexagonal phase has a lattice spacing of 3.33 Å. Furthermore, optical absorption spectra of CdS nanoparticles show a blue-shifted onset at 471 nm. Similarly, Ahamad et al. used a chemical precipitation method to produce cubic-phase CdS QDs and observed a similar blue shift due to the quantum size effect [97].

Jose et al. synthesized luminescent CdTe QDs via a heterogeneous reaction at 70 °C, using a less toxic cadmium source with a narrow size distribution [98]. Costa et al. used the seed-mediated growth approach to show that solution concentration has little effect on reflection-geometry in situ photoluminescence monitoring using this method [99]. They found that CdTe QDs synthesized in aqueous solution at 70, 80, and 90 °C exhibited a greater redshift with increasing synthesis temperature, due to rapid growth and a reduced bandgap. Chung et al. synthesized CdS_x_Se_1−x_ alloy QDs and found that changing the S:Se ratio in the core material can tune the band gap of CdS_x_Se_1−x_ nanocrystals from 1.96 to 2.32 eV, resulting in fluorescence color changes from red to green [100]. Also, ZnS shell coverage increased the PL quantum yield to 57%, caused a small redshift, and HRTEM confirmed highly crystalline, narrowly distributed particles for both CdS_x_Se_1−x_ core QDs and CdS_x_Se_1−x_/ZnS core/shell QDs.

Lead chalcogenide QDs (PbX, X = S, Se, and Te) are promising photovoltaic materials due to their affordable fabrication process, scalability, and tunable band gaps [101,102]. PbX QDs exhibit significant interdot wavefunction overlap and improved electronic coupling owing to their strong quantum confinement and large Bohr radii [102]. Yuan et al. synthesized PbS QDs using PbBr_2_ precursors, which resulted in smaller QDs due to stronger Pb-Br bonds [103]. This resulted in slower growth and smaller QDs than PbCl_2_ synthesis. In another study, Liu et al. [104] reported that the top-down method enables the synthesis of controlled PbS QDs with narrow dispersions (4.8% < σ < 7%) for use in optoelectronics applications. They found that PbS QDs exhibit uniform monodispersed, colloidal stability, and crystallinity, with a lattice spacing of 0.297 nm. They found that the observed red shifts of the 800–1000 nm excitonic peaks within 20 min confirm this method’s high reproducibility and controllability. Moreover, the rapid formation of PbS fragments during a 5 min ripening stage demonstrates that QD growth is independent of the oleic acid injection temperature and that QD size can be precisely controlled via the intermediate cracking step, enabling controllable synthesis.

**Figure 4:**
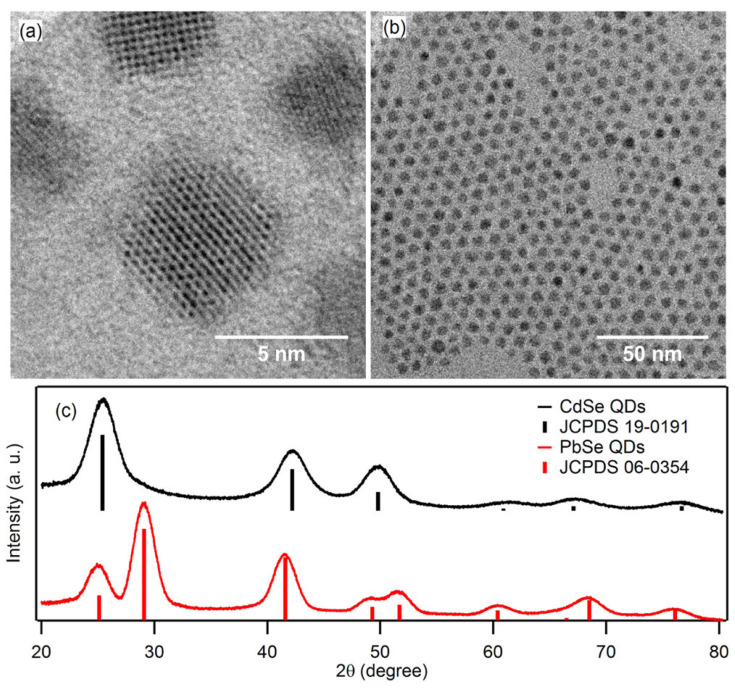
(**a**) HRTEM and (**b**) lower-resolution TEM images of resulting 4.7 nm PbSe QDs. (**c**) XRD patterns of 4.7 nm ion-exchanged PbSe QDs (red trace) and CdSe QDs (black trace), used as a starting material. Reprinted with permission from Ref [105]. Copyright 2014 American Chemical Society.

PbS QDs remain the primary focus in QD-PVs due to their air stability. However, PbSe QDs, which offer higher multiple excitation generation efficiency, suffer from severe ambient degradation during fabrication [105]. Zhang et al. reported that PbSe QDs with a wide size range and long-term air stability were achieved through a direct cation-exchange route that converts CdSe to PbSe QDs [105]. Moreover, elevated-temperature cation exchange creates defect-free, monodisperse PbSe QDs, as shown in Figure 4a,b, with the XRD image in Figure 4c confirming the absence of residual CdSe.

Pan et al. reported a simple method for making air-stable, monodisperse PbTe QDs that uses PbCl2 complexed with oleylamine (OLA) as the Pb precursor in a rapid reaction [106]. In this study, they adopted a temperature-driven PbCl_2_-OLA synthesis to produce PbTe QDs with tunable sizes (2.6–14 nm) and shape control. This provides an efficient technique for synthesizing monodisperse chalcogenide QDs. Badwi et al. used the successive atomic layer adsorption and reaction (SILAR) process to deposit Pb_1−x_Co_x_S (x = 0–0.4) ternary alloy QDs onto TiO_2_ nanoparticle electrodes and studied their characteristics by altering composition [107]. They reported that the absorption spectra of Pb_1−x_Co_x_S QDs on photoanodes exhibit a blue shift with increasing Co content due to the substitution of Pb by Co. Moreover, the band gap of Pb_1−x_ Co_x_S increases from 1.77 to 2.31 eV with increasing Co content, and the blue shift relative to the bulk is attributed to quantum confinement.

### 4.2. Carbon Quantum Dots

**Figure 5:**
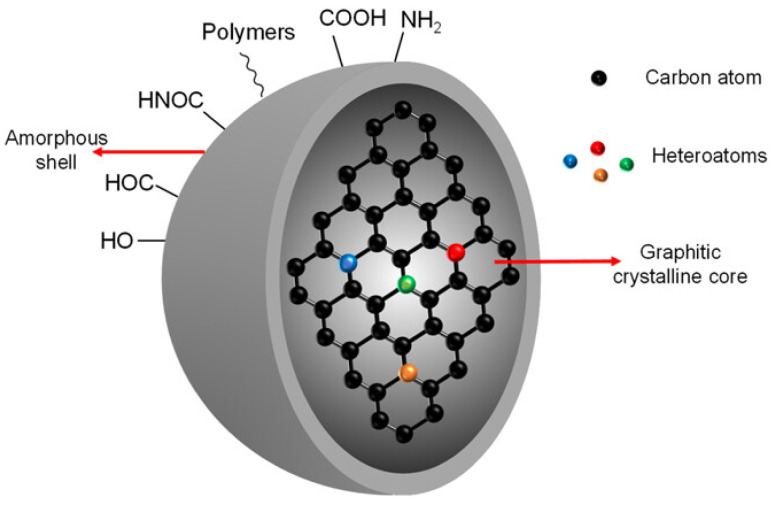
The general core–shell structure (left) [118] and chemical structure (right) of CQDs. Reprinted with permission from Ref [119]. Copyright 2013 Institute of Physics.

Carbon quantum dots (CQDs) are photoluminescent carbon-based nanomaterials. CQDs attracted some research interest due to their high solubility and biocompatibility, which are important for energy conversion and optoelectronics [108,109,110]. CQDs were accidentally discovered in early 2000 during the extraction of single-walled carbon nanotubes [109]. The improved fluorescent properties were then demonstrated using the surface passivation procedure [108]. CQDs are biocompatible and cost-effective, unlike conventional semiconductor QDs that contain heavy metals (Cd, Pd, or Hg) [111,112,113]. They are structurally quasi-spherical nanoparticles, typically smaller than 10 nm in diameter, and can be formed from crystalline sp^2^ graphitic cores or amorphous carbon aggregations, which produce a strong quantum confinement effect [114]. Their nanocrystalline form with amorphous carbon cores includes sp^2^-hybridized graphitic domains interlaced with sp^3^ diamond-like carbon, and is coated with numerous functional groups containing oxygen, such as carboxyl, hydroxyl, and carbonyl moieties [115,116]. These properties enhance aqueous solubility and enable chemically active regions for more passivation or hybridization with polymeric, organic, inorganic, or biological components. Figure 5 shows a schematic representation of the CQDs’ core cell and chemical structure [117].

Over the past 20 years, numerous top-down (such as laser ablation, electrochemical oxidation, and arc discharge) and bottom-up (such as pyrolysis, hydrothermal/solvothermal, and microwave-assisted) synthetic techniques have been developed, providing fine control over size, surface chemistry, and visual response [120]. These configurable characteristics result in unique optoelectronic properties, such as broad absorption spectra, tunable and excitation-dependent photoluminescence, up- and down-conversion emission, and superior photostability [121,122]. These attributes make CQDs useful in QDSCs, since they can act as interfacial modifiers, energy mediators, transport boosters, and defective passivators. Initial research on CQDs in solar cells began in the late 2000s, focusing on sensitized structures. However, such liquid-junction devices are low efficiency (≪1%) due to poor CQD light absorption and unfavorable interfacial charge transfer [110,123]. Recognizing the need for solid-state device integration, researchers are now focusing on using CQDs as active layers or interfacial dopants.

### 4.3. Perovskite Quantum Dots

Perovskite QDSCs are emerging as a promising photovoltaic technology that leverages the unique optoelectronic properties of metal halide perovskites and the benefits of quantum confinement effects [124]. Unlike their bulk thin-film counterparts, it uses QDs as discrete building blocks, which makes it easier to control composition, size, and surface chemistry while maintaining the defect-tolerant electronic structure of perovskite materials. Metal halide perovskites have garnered significant attention as promising photovoltaic candidates due to their tunable band gap [125], carrier migration capacity [126], and low exciton binding energy [127], etc. Since its first use in solar cells with an efficiency of 3.8% in 2009 [128,129], the progress has been significant and has reached efficiencies exceeding 26%, compared to those of silicon-based solar cells [129]. However, stability remains a significant constraint and is sensitive to temperature and humidity. Therefore, great effort has been made to extend the device’s lifespan by passivating defects or minimizing degradation. Even with such a strategy, structural stability could not be achieved due to the volatile and hygroscopic nature of the material and the limited number of scalable film deposition techniques [130,131,132].

To reduce problems associated with bulk perovskite materials, perovskite QDs were introduced and gained popularity. They offer advantages such as improved environmental stability, tunable energy levels and absorption spectra, high defect tolerance, and minimal electron–hole trapping [133,134]. Additionally, synthesizing and coating PQDs are less problematic, as they allow for precise control over size, thickness, and morphology compared to bulk perovskite films [135]. Moreover, the multiple exciton effect is helpful to exceed the Shockley–Queisser limit, so a higher theoretical efficiency is expected [136].

**Figure 6:**
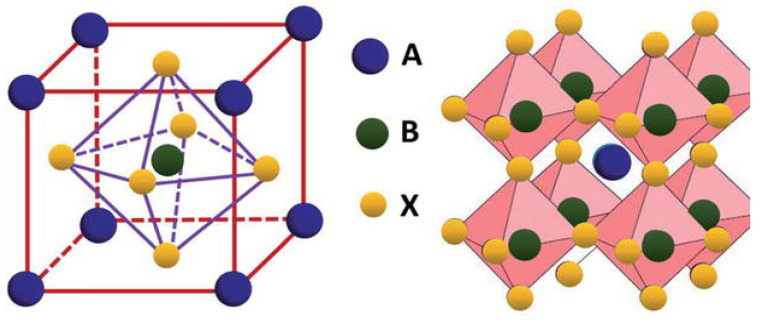
Structure of perovskite [140]. Reprinted with permission from ref. [140]. Copyright 2020 IntechOpen.

Lead halide perovskite QDs are promising photovoltaic materials due to their stable energy levels, suitable diffusion lengths, and tunable energy bands [137]. As illustrated in Figure 6, they have a chemical formula of ABX_3_, where A is usually an organic cation (for example, methylammonium (MA)) or a cation of an alkali metal (for example, Cesium), B is a cation from a positively charged ion with a +2 valency (for example, lead and manganese), and X is an anion of a halogen atom (for example, chlorine) bound to two cations [138]. Among them, perovskite crystals with a size of 2–10 nm are referred to as perovskite QDs [139]. Due to the restriction of electron motion within the perovskite lattice, quantum confinement enables tunable band gaps distinct from those of bulk perovskite systems.

Leads have been used in high-efficiency solar cells, but they cause toxicity problems. Additionally, they break down quickly when exposed to moisture and ultraviolet radiation. The structural stability of perovskite QDs is crucial to consider for device performance and longevity. In perovskite material, stability and formation of structure depend on two important dimensionless geometric parameters, i.e., the tolerance factor (*t*) and octahedral factor (*μ*), shown in Equations (7) and (8) [141],

(7) $$t=\frac{r_{A}+r_{X}}{\sqrt{2}\left(r_{B}+r_{X}\right)}$$

(8) $$\mu =\frac{r_{B}}{r_{X}}$$

where $r_{A}$, $r_{B}$, and $r_{X}$ are the ionic radii of the A-site cation, B-site cation, and halide anion, respectively. The tolerance factor (T) predicts structural stability and symmetry by evaluating how well the A-site cation fits into the space created by the BX_6_ octahedron. The octahedral factor (*μ*) indicates the stability of the BX_6_ octahedron by relating the size of the B-site cation to the X-site anion. It determines whether an octahedron can be formed within the structure without significant strain.

For halide (X = F, Cl, Br, I) perovskites, 0.81 < *t* < 1.11 and 0.44 < *μ* < 0.90 [142]. For *t* between 0.89 and 1, the structure is cubic. However, if this value is lower, a tetragonal or orthorhombic structure is expected. Although there is a restriction, the structures are often interchangeable upon heating.

### 4.4. Electron-Transport Layer and Hole-Transport Layer

Efficient charge extraction and transport are important to the performance of solid-state QDSCs [13,143,144]. However, the overall device efficiency is strongly governed by the properties of the electron-transport layer (ETL) and hole-transport layer (HTL) incorporated in the device architecture.

The interface between the QD active layer and the transport layer plays a crucial role in determining device efficiency. Defective interfaces can introduce trap states that serve as recombination centers, thereby reducing charge-carrier lifetimes. To address these challenges, strategies such as surface passivation, defect control, and thickness optimization have been widely employed. Simulation studies indicate that careful tuning of ETL and HTL properties, including doping concentration, layer thickness, and defect density, can significantly enhance power conversion efficiency, in some cases exceeding 13–16% in PbS QDSCs [145].

Furthermore, environmentally stable and low-temperature processable materials, such as SnO_2_ and NiO, have gained attention for their potential in sustainable photovoltaic technologies [146]. Overall, the careful selection and optimization of ETL and HTL materials remain essential for improving carrier selectivity, minimizing recombination losses, and enhancing both the efficiency and stability of quantum dot solar cells.

#### 4.4.1. Electron-Transport Layers

The electron-transport layer (ETL) facilitates the extraction and transport of photoexcited electrons from the QD active layer to the electrode (often indium tin oxide or fluorine-doped tin oxide, ITO/FTO). Ideal ETLs must offer conduction bands with the lowest unoccupied energy levels below that of the QDs. ETLs must also offer high electron mobility, minimal defects to avoid electron trapping, and high thermal and chemical stability. Examples of common ETL materials are TiO_2_ (Titanium Dioxide), ZnO (Zinc Oxide), SnO_2_ (Tin Oxide), In_2_O_3_, and organic semiconductors [147,148].

The concept of electron-selective transport layers in thin-film photovoltaics was popularized in dye-sensitized solar cells (DSSCs), where mesoporous TiO_2_ served as the electron-transport scaffold [149]. The adaptation of this ETL/HTL architecture to QD-based systems began gaining traction in the early 2000s, with the pioneering incorporation of PbS QDs into solution-processed photovoltaics [150]. The use of TiO_2_ as an ETL was adopted directly from the DSSC architecture, while ZnO emerged as a competitive alternative in the late 2000s owing to its higher intrinsic electron mobility (TiO_2_~10^−4^–1 cm^2^/V·s, ZnO~1–200 cm^2^/V·s) and compatibility with low-temperature processing. SnO_2_ gained broader attention after approximately 2015, largely driven by its success in perovskite solar cells, which were later translated into QDSC architectures. For efficient device operation, an ideal ETL should exhibit proper band alignment with the conduction band of the QDs [143,147,151], high electron mobility, low interfacial recombination losses [151,152,153], and good thermal and chemical stability.

Several studies have demonstrated the critical role of ETLs in improving charge extraction and device performance. For example, device simulations comparing MgZnO (MZO) and TiO_2_ electron-transport layers in PbS CQD solar cells revealed that TiO_2_ provides superior device performance due to its higher electron mobility (~20 cm^2^ V^−1^ s^−1^). Replacing MZO with TiO_2_ improved the fill factor from 60.8% to 63.5% and increased efficiency from 9.43% to 9.87%. Optimization of ETL donor doping showed that a doping density of approximately 5 × 10^17^ cm^−3^ enables efficiencies approaching 14% [154].

Similarly, simulation studies have identified that among the investigated ETLs (CdS, C60, ZnSe, CeO_2_, Nb_2_O_5_, ZnO, TiO_2_, IGZO, PCBM, WO_3_, and WS_2_), WS_2_ exhibited the best performance due to its favorable conduction band alignment and high carrier mobility, which enhanced electron extraction from the PbS-TBAI absorber layer. Replacing TiO_2_ with WS_2_ improved the short-circuit current density from 28.50 mA cm^−2^ to 33.93 mA cm^−2^, leading to significant improvements in photovoltaic parameters. The optimized device structure ITO/WS_2_/PbS-TBAI/MoO_3_/Au achieved a power conversion efficiency of approximately 23.29%, with *V_oc_* ≈ 0.879 V, *J_sc_* ≈ 33.93 mA cm^−2^, and FF ≈ 78%, demonstrating that WS_2_ is a promising ETL material for high-efficiency PbS quantum dot solar cells (Figure 2) [155].

Alternative ETL materials, such as SnO_2_, have also been investigated. Vijayaraghavan et al. [156] developed a device using low-temperature processed SnO_2_ ETL, while eliminating the HTL entirely. By engineering ligand gradients within the PbS absorber using TBAI and EDT ligands, the device achieved efficient hole transport without a dedicated HTL and reached efficiencies of 11.44%, demonstrating the potential of simplified device architectures. We also want to bring awareness that CdS QDs [157], 2D materials [158], and ZrO_2_ [159] were being used as non-conventional ETL materials in SCs, as summarized in Table 1.

**Table 1:** Common ETL materials in QDSCs.

ETL Material	Key Properties	Advantages	Limitations	References
**TiO_2_**	Wide bandgap (~3.2 eV), strong electron mobility	Excellent band alignment with PbS QDs, high transparency, stable	Slow electron transport in some morphologies	[155,160,161]
**ZnO**	High electron mobility, solution processable	Low-temperature fabrication, efficient electron extraction	Interface traps may cause recombination	[154,162,163]
**SnO_2_**	High conductivity, wide bandgap	Low-temperature processing (<150 °C), high stability	Interface defects possible	[156,164]
**CdS QDs**	Tunable conduction band via quantum confinement	Precise band alignment with PbS QDs	Potential toxicity concerns	[157]
**2D Materials (MoS_2_, MXenes, Graphene)**	High mobility, tunable band structure	Reduced recombination, improved interface contact	Still emerging technology	[158]
**ZrO_2_**	High transparency, chemical stability	Eco-friendly alternative ETL	Lower conductivity than other oxides	[159]

#### 4.4.2. Hole-Transport Layers

HTL is responsible for extracting holes from the QD active layer and transporting them to the counter electrode (often Au or Ag). An efficient HTL must have the highest occupied valence-band level, lower in energy than that of the QDs. The HTL should exhibit high hole mobility, low defect density to prevent electron back-transfer and recombination, and environmental and chemical stability. Some common HTL materials include MoO_3_ (Molybdenum Trioxide) and organic/polymeric compounds (PTAA, P3HT, PEDOT:PSS) [153].

PEDOT (poly(3,4-ethylenedioxythiophene)) was first synthesized by Bayer AG in 1988, with the processable PEDOT:PSS composite form developed in 1990 [165]. It has become one of the most widely used polymeric HTLs in solution-processed photovoltaics. spiro-MeOTAD was first synthesized in the late 1990s as a small-molecule HTL designed for high morphological stability [166], and was later adapted into solid-state solar cell architectures. In 2010–2015, inorganic HTLs, such as MoO_3_ and NiO, gained momentum, driven by their superior environmental stability and better energy-level alignment with QD valence bands. An efficient HTL must satisfy several requirements: proper valence-band alignment with the QD layer [167], high hole mobility and conductivity, effective suppression of electron back-transfer, and environmental and chemical stability [148].

**Figure 7:**
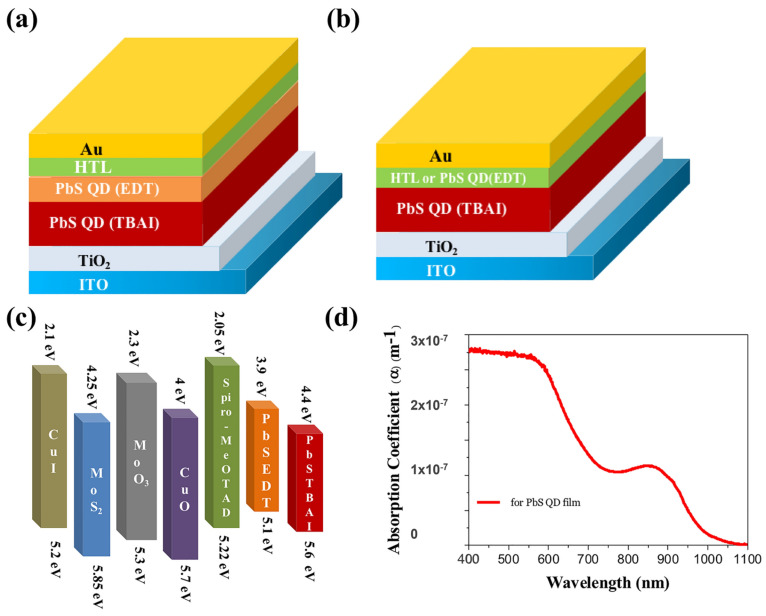
Schematic structure of (**a**) architecture—1(ITO/TiO_2_/PbS-TBAI/PbS-EDT/HTL/Au) and (**b**) architecture—2 (ITO/TiO_2_/PbS-TBAI/HTL/Au). (**c**) Energy band diagram of PbS QDs solar cell with different HTLs. (**d**) Wavelength-dependent absorption coefficient of PbS QD thin film [145]. Reprinted with permission from ref. [145]. Copyright 2023 Springer Nature.

Numerical simulations investigating various HTLs (including CuI, MoS_2_, MoO_3_, CuO, and spiro-MeOTAD) revealed that the selection significantly affects the performance of PbS QDSCs (Figure 7). MoO_3_ exhibited the best performance due to favorable band alignment with the PbS-TBAI absorber layer and efficient hole transport. The optimized device structure ITO/TiO_2_/PbS-TBAI/MoO_3_/Au achieved a power conversion efficiency of 16.43%, with *V_oc_* ≈ 0.783 V, *J_sc_* ≈ 28.50 mA cm^−2^, and *FF* ≈ 73.61% [145].

Organic HTLs, such as PEDOT:PSS and spiro-MeOTAD, have also been widely used. PEDOT:PSS is particularly attractive due to its excellent film-forming ability, high hole mobility, and compatibility with solution-processed devices. However, its sensitivity to moisture can affect its long-term stability [161]. In contrast, inorganic HTLs, such as NiO, CuO, and CuSCN, offer improved environmental stability and long-term durability. These materials exhibit suitable valence band alignment with quantum dot absorbers and can improve hole transport without the stability limitations associated with organic HTLs [164].

To reduce device complexity and improve stability, researchers have also explored HTL-free architecture. For example, Taheri-Ledari et al. [168] demonstrated a high-performance HTL-free perovskite solar cell using a co-doped TiO_2_ ETL. The incorporation of Mn^2+^ and Ni^2+^ dopants improved charge mobility and energy-level matching, enabling efficient electron transport and direct hole extraction at the perovskite/Au interface. This simplified device achieved a power conversion efficiency (PCE) of 18.43%, demonstrating the feasibility of eliminating the HTL layer without sacrificing performance.

#### 4.4.3. Emerging Transport Layers: Two-Dimensional Materials

Two-dimensional materials have recently emerged as promising candidates for advanced charge transport layers in QDSCs. Materials such as graphene, MXenes, and MoS_2_ have attracted significant attention due to their high carrier mobility, tunable band structures, and excellent interfacial compatibility with quantum dots. These materials can serve as efficient ETLs by improving charge transport and reducing interfacial recombination losses [158].

Similarly, graphene oxide, MoTe_2_, and NiO have been explored as HTLs due to their efficient hole extraction and strong chemical stability. Among these materials, NiO has demonstrated effectiveness in p–i–n device architectures, offering wide band gaps and improved resistance to degradation. Some of these HTLs and their properties are summarized in Table 2.

## 5. Devices Based on Various QDs

### 5.1. Cd-Based Quantum Dot Solar Cells

Liu et al. [172] used a solution process and CdCl_2_-based sintering to fabricate inverted CdTe-CdSe nanocrystal solar cells with a (ITO/ZnO/CdSe/CdTe/Au) structure. While ITO/ZnO films are highly transparent, CdSe layers exhibit strong absorption between 400 and 700 nm, which increases with thickness. Because a thick CdSe layer reduces light transmission to the CdTe absorber, optimizing its thickness is critical for efficient heterojunction operation. They also reported that the ZnO layer enables the formation of a uniform and compact CdSe NC film, which prevents current shunting, and that moderate annealing (320–350 °C) yields high efficiency (PCE of 5.81%), whereas overheating (>360 °C) induces shunting and reduces performance.

Yang et al. [173] incorporated low-dose CdSe QDs to improve the performance of ternary PTB7-Th: PC71BM-based devices. They found that CdSe QD incorporation in PTB7-Th:PC_71_BM BHJ solar cells suppresses recombination losses, resulting in an 11% improvement in PCE over the binary system. They mentioned that with 5 wt% CdSe QDs, the power conversion efficiency increases to 9.57%, along with improved *J_sc_*, *V_oc_*, and FF. Beyond this wt% concentration, excessive QDs degrade film morphology, lowering the fill factor and limiting device performance.

**Figure 8:**
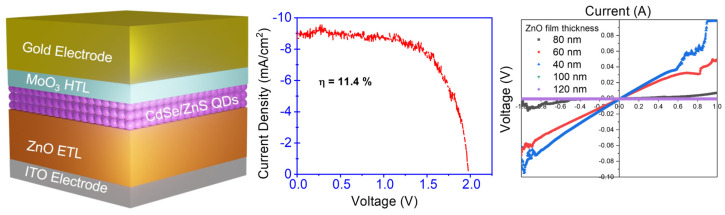
A schematic illustration of the QDSCs with core–shell CdSe/ZnS QDs as the active layer. A PCE of 11.4% was reported for ZnO 80 nm thick, with enhanced semiconducting properties. Reprinted with permission from ref. [174]. Copyright 2025 American Chemical Society.

More recently, Achaya et al. suggested that improving the quality of ETLs could enhance the efficiency of ITO/ZnO/core–shell CdSe/ZnS QDs/MoO_3_/gold devices [174]. As illustrated in Figure 8, a PCE of 11.4% was obtained (middle panel). The *V_oc_* of the devices is approaching the theoretical limit of ~2 V for ZnO-based devices. The ZnO film exhibits greater semiconducting behavior at the optimum thickness of 80 nm versus other film thicknesses shown in the right panel.

### 5.2. Pb-Based Quantum Dot Solar Cells

#### 5.2.1. Overview of Devices and Interface Losses

Lead-based quantum dot solar cells (Pb-QDSCs) have emerged as a promising photovoltaic technology [13,164,175,176]. However, despite their rapid progress, Pb-QDSCs still face significant challenges, limited by non-radiative recombination, interfacial losses, and long-term instability under operating and ambient conditions [34,145].

Heterojunctions were used to reduce interface loss. Kim and Ma highlighted the ability of PbS QDs to achieve size-tunable band gaps, with optimal band gaps around 1.1–1.3 eV [144]. The article emphasized PbS’s suitability for multijunction architectures and the exploitation of advanced carrier dynamics, such as multiple exciton generation (MEG) and hot-electron extraction, which could allow efficiencies to surpass the Shockley–Queisser limit of 31% [144]. A study by Ip et al. [151] provided a deeper look into PbS-based thin-film heterojunction solar cells, with an emphasis on device architecture optimization. They constructed PbS/CdS planar junctions and demonstrated the critical role of conduction- and valence-band alignment in facilitating efficient charge extraction. Their results showed that by tuning PbS QD sizes (~2.7 nm, Eg ≈ 1.57 eV) and employing optimized CdS layers (~70 nm), devices could achieve PCEs of 3.3%, with open-circuit voltages as high as 0.65 V, reflecting progress in interface engineering and film uniformity [177]. Another study demonstrated that carefully controlling the interfacial band alignment between the lead chalcogenide QD layers and transport layers significantly improves both efficiency and long-term stability. By introducing energy-aligned buffer layers and optimizing carrier extraction, they reported a notable boost in PCE up to 9.1%, while suppressing charge recombination and enhancing device durability [67].

Bohm et al. [178] explored PbTe QDs as a viable alternative to PbS QDs. PbTe QDs have smaller band gaps, allowing the harvesting of red to near-IR light into energy. They achieved external quantum efficiencies (EQEs) up to 80% in the near-infrared (1200–1400 nm), demonstrating the potential of PbTe QDs to extend the spectral response of QDSCs. However, overall PCEs remained modest (~2%), indicating the need for additional interface and passivation strategies.

#### 5.2.2. Doping and Interface Passivation

Doping attempted to suppress interface losses. For example, doping of PbS QDs with Hg^2+^ ions, which strengthens Pb–S bonds, improves electron injection into TiO_2_, and reduces recombination. This has led to *J_sc_* of 30 mA/cm^2^ and PCE exceeding 5.5%. Femtosecond studies have confirmed faster carrier injection and slower recombination as the primary benefits [179]. Another strategy involves forming heterojunctions with CdS film, achieving open-circuit voltages up to 0.65 V and PCEs around 3.3%, using ~2.7 nm PbS QDs and ~70 nm CdS films. Performance relies heavily on optimized band alignment at the interface [180]. Doping CdS QDs with 2% Pb significantly improves light absorption and photovoltaic efficiency when deposited on TiO_2_ substrates, using the SILAR method. This doping creates mid-gap states that improve carrier lifetimes and reduce recombination, achieving a power conversion efficiency (PCE) of 1.19% and a short-circuit current density (*J_sc_*) of 3.76 mA/cm^2^, without altering the morphology of the QD [181].

Interface passivation can be performed by capping the QDs with ligands. Speirs et al. [182] conducted a systematic temperature-dependent study on PbS QDSCs to understand limitations in open-circuit voltage (*V_oc_*) and fill factor (FF). Their devices, combining TBAI and EDT ligands, showed increased *V_oc_* and FF at lower temperatures, with a peak PCE of 10.3% at 230 K. They linked this improvement to a reduction in reverse saturation current rather than bandgap changes, and proposed doping concentration optimization as a more effective route to enhance *V_oc_* at room temperature. On the other hand, there is substantial interest in passivating Pb-based QDs by incorporating them with perovskite. Hosokawa et al. [183] introduced a solution-processed intermediate-band solar cell (IBSC), using PbS QDs embedded in a methylammonium lead bromide perovskite matrix. This architecture enabled two-step photon absorption (TSPA) and achieved a dense, strain-free QD distribution. While the PCE showed a slight reduction due to charge-transfer dynamics, the cells exhibited functional intermediate-band behavior and demonstrated successful near-infrared photon harvesting at room temperature.

Another issue with Pb-based QD SCs is environmental and operational stability. PbS QD films are vulnerable to degradation by oxygen, moisture, and thermal stress, so several groups have developed hybrid passivation schemes to stabilize the absorber while also preserving favorable charge transport. In pursuit of improving both efficiency and stability in PbS QD solar cells (QDSCs), Albaladejo-Siguan et al. introduced a novel passivation approach using a triple-cation perovskite (Cs^+^/MA^+^/FA^+^) layer [184]. This strategy not only minimized interfacial trap states but also improved energy-level alignment between the PbS QD layer and the electron-transport layer. By engineering a graded energy landscape through this perovskite capping layer, the authors achieved a power conversion efficiency (PCE) of 11.57% and enhanced air stability, with devices maintaining over 90% of initial performance after 1000 h in ambient conditions. Importantly, the triple-cation perovskite layer functioned as a multifunctional passivation and buffer layer, simultaneously enhancing photocarrier transport and reducing recombination losses. This work highlights the potential of perovskite–QD hybrid interfaces to push PbS QDSC performance closer to commercialization [184].

Building upon integrating PbS with perovskite, Ding et al. (2022) demonstrated a record PCE of 15.45% in PbS QDSCs by engineering all three major interfaces: QD/QD, QD/ETL, and QD/HTL [185]. This was achieved through a synergistic approach involving (i) ultrathin perovskite shell growth for internal passivation, (ii) a PMMA-PCBM layer to balance ETL interaction, and (iii) a PMMA–graphene oxide buffer to enhance hole extraction and reduce recombination. Further innovation explored carbon-coated lead halide perovskite QDs, in which a thin carbon shell served as a barrier to moisture and oxygen ingress, thereby improving both stability and performance. The coating not only reduced surface trap states but also facilitated improved charge-carrier extraction across interfaces [186].

Complementary to these efforts, Guo et al. designed p-type graphene QDs to mitigate interfacial charge imbalances, enhancing charge transfer at the HTL, improving FF, and reducing hysteresis in Pb-based devices [187]. Kumar et al. [145] employed SCAPS-1D numerical simulations to explore optimal device configurations, using PbS-TBAI as the absorber material. They found a simplified architecture—ITO/TiO_2_/PbS-TBAI/HTL/A outperformed traditional multilayer designs by minimizing interface defect densities, particularly at the TiO_2_/PbS interface. These simulations not only confirmed the potential for higher power conversion efficiency (PCE) but also highlighted the sensitivity of device output to interfacial recombination. In an ambitious effort to push the boundaries of Pb-QDSC efficiency, Hasnain et al. (2025) [94] reported a record-breaking simulated PCE of 32.9% by leveraging device-architecture engineering through SCAPS-1D modeling. Their approach included optimizing layer thicknesses, doping concentrations, and defect densities, thereby presenting a roadmap for high-efficiency lead-based solar cells that are potentially competitive with tandem architectures.

To sum up, PbS QDs were initially explored in sensitized configurations using TiO_2_ scaffolds, achieving modest power conversion efficiencies (PCEs) of ~1–2% due to poor interfacial charge transport and rapid recombination [181]. Early improvements came from heterojunction designs with CdS and optimized QD sizes, enhancing open-circuit voltages and pushing PCEs toward 3–5% [179,180]. A major milestone was achieved through interface engineering and doping strategies—such as Hg^2+^ doping and valence band alignment—which enabled faster electron injection and reduced recombination, raising PCEs above 5.5%. Subsequent advances involved hybridizing PbS QDs with perovskite shells, carbon coatings, and buffer layers, leading to dramatic improvements in both performance and stability. Notably, ref. [185] reported an >15% PCE by simultaneously engineering three key interfaces in the device. Complementary efforts, including triple-cation perovskite passivation [184] and p-type graphene QD buffers [187], further mitigated non-radiative losses. Most recently, simulation-driven optimization has suggested that efficiencies up to 32.9% are theoretically achievable with idealized architectures [94].

### 5.3. Solar Cells Based on Carbon Quantum Dots (CQDs)

**Figure 9:**
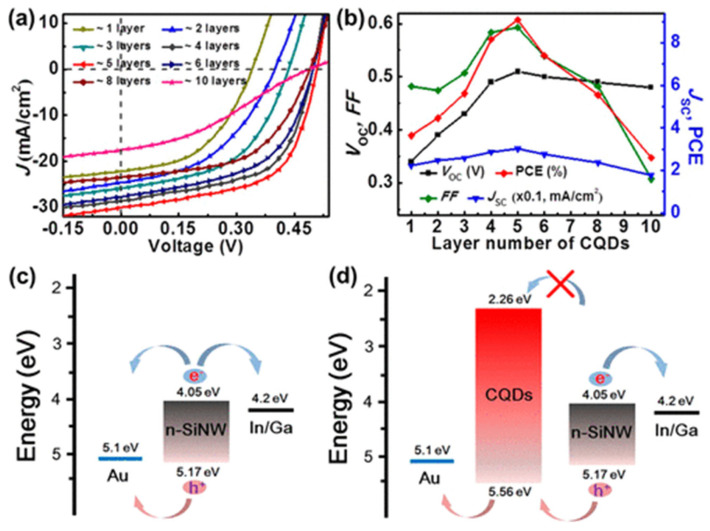
(**a**) The photovoltaic characteristics dependence of CQD heterojunction devices/SiNW array over the thickness of CQDs. (**b**) The plots of *V_oc_*, *J_sc_*, *FF*, and PCE as functions of the CQD layer number. Energy band diagrams of the Au/SiNW array Schottky junction, (**c**) and the SiNW array/CQD heterojunction (**d**). Reprinted with permission from ref. [188]. Copyright 2014 American Chemical Society.

Graphene-derived QDs were initially added to the active layer of bulk-heterojunction (BHJ) organic solar cells [110]. However, the first solid-state CQD solar cell was reported in 2014. Xie et al. formed a core–shell nanostructure by directly drop-coating a CQD solution over vertically oriented silicon nanowire (SiNW) arrays [188]. The study yielded devices with a PCE of 9.10%, as shown in Figure 9.

#### 5.3.1. Carbon Quantum Dots in Electron- and Hole-Transport Layers

Carbon quantum dots (CQDs) serve as ETLs in organic and perovskite solar cells due to their photostability, low toxicity, strong electron-extraction capability, and ease of bandgap tuning. Two main approaches to using CQDs as ETLs have been investigated by researchers: (i) replacing current ETLs entirely, and (ii) combining with conventional ETLs to improve performance. Using chemical vapor deposition (CVD), Yan et al. fabricated CQDs as ETL in a solution-processed organic solar cell with the device structure ITO/PEDOT:PSS/active layer/CQDs (ETL)/Al [189]. PCEs of 3.11%, 6.85%, and 8.23% were obtained for the P3HT:PC61BM, PTB7:PC61BM, and PTB7-TH: PC71BM devices, respectively. By reducing interfacial resistance, CQDs improved electron injection, device performance, and thermal stability in the ETL.

**Figure 10:**
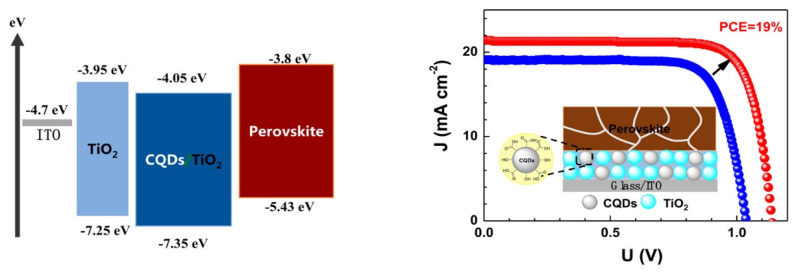
Band diagram (**left**), current density-potential (J-U) curve (**right**) of a perovskite solar cell with TiO_2_ ETL (blue curve) and with CQDs/TiO_2_ composite ETL (red curve) [190]. Reprinted with permission from ref. [190]. Copyright 2017 American Chemical Society.

Li et al. [190] later improved the PCE to ~19% by using CQDs/TiO_2_ composite layers as ETLs in planar n–i–p heterojunction perovskite solar cells (Figure 10). At the TiO_2_/perovskite interface, the CQDs facilitated electron transport and charge extraction/injection, which improved the open-circuit voltage (*V_oc_*) and short-circuit current density (*J_sc_*). Later, Zhu et al. used CQD-doped PCBM as the ETL in a planar heterojunction perovskite solar cell and observed a gradual improvement in PCE from 16.1% to 18.1% relative to the pure PCBM-based device [191]. Doping with CQDs improves the conductivity of the ETL and prevents I^−^ diffusion into the Ag electrode, thereby suppressing fullerene dimerization and enhancing device stability [191].

Park et al. fabricated QCDs with NH_2_ ligands via microwave-assisted hydrothermal treatment of Neutral Red and ethylenediamine [192]. CQDs were doped into the PEI layer of organic solar cells (ITO/CQD-doped PEI/PTB7-Th: PC71BM/MoO_3_/Ag). The CQD-doped PEI layer significantly lowered the work function of ITO, which strengthened electron transport. The results showed that the optimized PCE with 2% CQDs was 9.468%, compared to 8.549% for the pristine PEI-layer-based device. The increase in efficiency was attributed to (i) improved electron transport because of CQD doping, and (ii) an enhanced exciton dissociation probability triggered by an increased internal electric field.

CQDs also showed promising results when used as the HTL in methylammonium lead iodide (MAPI) perovskite solar cells [193]. PCEs of 3.00%, open-circuit voltages (*V_oc_*) of 0.515 V, short-circuit current densities (*J_sc_*) of 7.83 mA/cm^2^, and fill factors (FF) of 74% were all attained through the devices. Although it did not perform as well as traditional HTLs, such as spiro-OMeTAD, the study showed that CQDs can efficiently promote hole transit. These results highlight the promise of CQDs as an affordable and adaptable substitute in perovskite solar cells, generating ongoing interest in their broader use in perovskite and organic solar technologies to boost productivity and lower production costs [193].

Duong et al. incorporated nitrogen-doped CQDs and oxidized CQDs into the PEDOT: PSS layer to enhance hole transport in organic solar cells [194]. The addition of N-CQDs resulted in a PCE of 8.57%, which is higher than the 8.17% achieved with O-CQDs and the 7.26% for devices without CQDs. The addition of N-CQD improved charge extraction, lowered resistance, and raised work function, all of which contributed to higher device efficiency. The higher interaction between PEDOT and PSS with N-CQDs significantly enhanced the device performance.

#### 5.3.2. Recent Device Performance with Carbon Quantum Dots

Based on earlier discoveries, researchers continue to explore applications of CQDs beyond ETLs and HTLs for solid-state perovskite solar cells (PSCs). For example, A-CQDs (acetone-derived) and CA-CQDs (citric acid-derived) were incorporated into the perovskite precursor solution [195]. The A-CQDs improved the perovskite film morphology, crystallinity, and enhanced charge transport, leading to larger grain sizes and reduced recombination losses. As a result, A-CQD-modified PSCs exhibited a power conversion efficiency (PCE) of 13.28%, compared with the virgin device’s 10.50%. On the other hand, CA-CQDs reduced device performance by negatively affecting carrier recombination. Additionally, the A-CQD-modified PSCs demonstrated enhanced long-term stability, maintaining over 90% of their initial efficiency. This study demonstrates how CQD additives can enhance the stability and efficiency of solid-state perovskite solar cells.

To sum up, CQDs enhance PSC performance. When CQDs were integrated into the ETL, the device’s PCE rose from 22.31% to 24.48% [196]. In this arrangement, the module’s structure, ITO/PEDOT: PSS/Perovskite/CQDs-ETL/Ag, benefits from CQDs’ ability to fill gaps in the silver nanowire (Ag NW) network, thereby elevating conductivity and enabling more efficient charge transfer. This led to improved charge collection and interface quality. Likewise, recent research demonstrated the use of CQD in both ETL and HTL, resulting in a PCE of 22.5%. This twofold integration facilitated charge transfer and exciton dissociation, demonstrating the potential of CQDs as multifunctional additives [197]. Despite promising progress, significant obstacles remain in CQD-integrated solar cells, including the scalability of CQD synthesis and long-term stability under environmental stress [198]. The consistency of CQD diffusion across layers can affect device uniformity, so interface compatibility between CQDs and other materials should be addressed to reduce recombination losses.

### 5.4. Solar Cells Based on Perovskite Quantum Dots (PQDs)

The commonly used PQDs for solar cells are MAPbX_3_, FAPbX_3_, and CsPbX_3_. However, MA-based PQDs suffer from stability issues due to their low formation energy [199]. On the other hand, Cs- and FA-based perovskites are metastable at room temperature, and their stability can be enhanced by reducing their size to the nanometer scale. Swarnkar et al. [200] synthesized α-phase CsPbI_3_ QDs with a diameter of around 10–15 nm using oleic acid and oleyl amine ligands, and purified them with methyl acetate to remove excess precursor. They found that these QDs remained stable for months under ambient conditions. They first reported that α-CsPbI_3_ PQDSCs reached an efficiency of 10.8%, with a stabilized power output and a high open-circuit voltage (*V_oc_*) of 1.23 V, which outperforms the CsPBX_3_ thin-film device (PCE of 9.8% [201]). This demonstrated the potential of using PQDs in solar cells. The optical properties of PQDs were retained after storing in ambient conditions for 60 days.

#### 5.4.1. Progress in Perovskite Quantum Dot Solar Cells (PQDSCs)

Sanehira et al. used formamidinium iodide (FAI) in ethyl acetate as an A-site cation halide salt (AX) treatment for CsPbI_3_ QD films, which increased charge mobility to 0.5 cm^2^ V^−1^ s^−1^ [135]. This treatment led to a PCE of 13.43%. The study highlighted the significance of post-deposition ligand exchange strategies and demonstrated that careful surface modification can significantly enhance device performance by improving charge transport while maintaining surface passivation. This work introduced the concept of AX-salt treatment, which becomes fundamental to subsequent PQDSC device development.

Zhao et al. introduced the concept of perovskite–perovskite internal heterojunctions, where films composed of stacked CsPbI_3_/Cs_0.25_FA_0.75_PbI_3_ QD layers, which created favorable band alignments for charge separation [202]. They used a layer-by-layer deposition technique to form CsPbI_3_ and Cs_1−x_FA_x_PbI_3_ QD layers, creating a compositional gradient. This enabled efficient charge separation by driving electrons toward the FA-based (lower bandgap) layer and holes toward the CsPbI_3_ (higher bandgap) layer, thereby improving charge-carrier collection. The device architecture consisted of a TiO_2_ ETL, a spiro-OMeTAD HTL, and an Al electrode. Through this design, the solar cells achieved a PCE of 15.52%, a significant improvement over previous QD solar cell architectures, owing to enhanced charge-carrier dynamics and reduced recombination.

**Figure 11:**
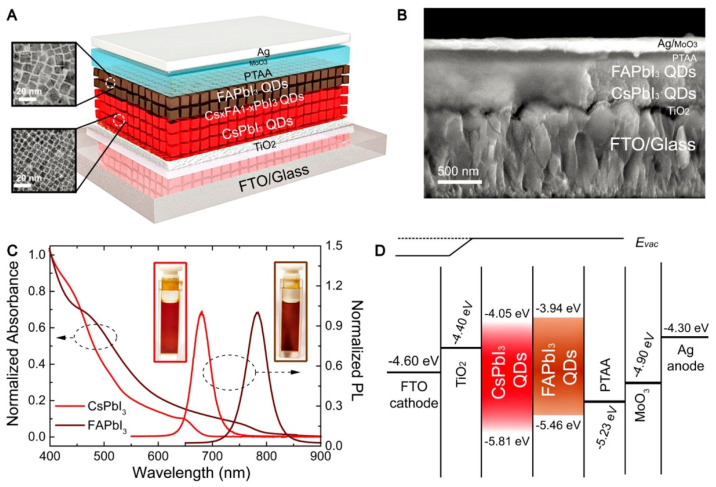
(**A**) A schematic illustration of the SCs with bi-layer PQDs. (**B**) A cross-sectional SEM image of the device. (**C**) The UV–vis and PL spectra of respective QDs. (**D**) An energy band diagram of MeOAc-treated QD films and the corresponding SCs [203]. Reprinted with permission from Ref. [203]. Copyright 2017 American Chemical Society.

Following a similar approach, Li et al. developed a high-efficiency PQDSC with a 15.6% PCE by constructing a bilayer α-CsPbI_3_/FAPbI_3_ QD structure [203]. The device architecture (Figure 11) consisted of a glass/ITO/PEDOT:PSS/α-CsPbI_3_/FAPbI_3_ QDs/PCBM/Ag configuration, in which α-CsPbI_3_ and FAPbI_3_ QDs, with distinct band gaps, enabled broader absorption and graded energy-level alignment, thereby enhancing charge-carrier extraction and transport. Methyl acetate (MeOAc) was used to partially remove the native ligands (oleylamine and oleic acid) on the QDs. The bilayer structure improved device efficiency and enhanced ambient stability, with the FAPbI_3_ layer protecting the α-CsPbI_3_ layer from degradation. The device achieved a *J_sc_* of 17.26 mA/cm^2^, a *V_oc_* of 1.22 V, and a fill factor (FF) of 74%, outperforming single-material QD devices.

The key protocol for fabricating PQDSCs is ligand-exchange. Hao et al. developed mixed-cation Cs_1−x_FA_x_PbI_3_ QDs, stabilized through ligand-assisted cation exchange [204]. These QDs were formed by blending two cations, cesium (Cs) and formamidinium (FA), thereby improving the stability and performance of perovskite solar cells. The device structure included layers, such as Glass/ITO/SnO_2_, the Cs_1−x_FA_x_PbI_3_ QD layer, spiro-OMeTAD as the hole-transport layer, and gold (Au) as the top electrode. The devices achieved a PCE of 16.6%, with a voltage (*V_oc_*) of 1.17 V, a short-circuit current density (*J_sc_*) of 18.3 mA/cm^2^, and a fill factor (FF) of 78.3%. They demonstrated improved stability, with efficiency remaining at 94% after 600 h of continuous illumination. The mixed-cation approach helped to reduce phase segregation—a common issue in perovskite materials— and enhanced charge extraction and carrier transport, making these solar cells more efficient and durable.

Jia et al. demonstrated an antisolvent-assisted in situ cation exchange process to produce uniform FA_x_Cs_1−x_PbI_3_ QDs with controlled compositions [205]. By systematically testing nine different compositions (x = 0 to 0.57) with 20 devices each, they identified FA_0.36_Cs_0.64_PbI_3_ as the optimal, achieving 17.29%. This composition showed improved light absorption (up to ~720 nm), better structural stability (tolerance factor = 0.87), and efficient charge transport. Excessive FA content (x ≥ 0.57) led to poor performance (~12% PCE) due to QD agglomeration. The planar ITO/SnO_2_/PQDs/spiro-OMeTAD/Ag devices benefited from an efficient ligand-replacement method that avoided the addition of extra-long-chain molecules, resulting in broader absorption, a more uniform energy landscape, and aligned crystallographic packing. The optimized devices maintained an efficiency of around 80% after 240 h under ambient conditions and 200 h under continuous illumination, compared to only around 15% and 66% for pure CsPbI_3_ devices, highlighting the importance of composition optimization for both performance and stability.

Aqoma et al. achieved a PCE of 18.1% for PQDSCs by improving the chemical treatment method [206]. The researchers worked with formamidinium lead triiodide (FAPbI_3_) QDs. A challenge with these materials is the need for long ligand molecules (oleic acid and oleylamine) to stabilize the QDs, but the ligands reduce current flow in the finished devices. They tested three different chemical treatments: (1) the conventional method using lead nitrate in methyl acetate (PQD-PbNO_3_), which only partially removed the long molecules and achieved 13.86% efficiency, (2) the use of formamidinium iodide in isopropanol (PQD-FAI) to remove the long ligand molecules and transformed the QDs into an inactive crystal phase for achieving 15.05% efficiency, and (3) a new approach using methylammonium iodide in isopropanol (PQD-MAI), which both removed the long molecules and prevented crystal degradation by incorporating methylammonium ions into the structure. This created a mixed-cation composition (FA_0.42_MA_0.58_PbI_3_) that was both highly conductive and structurally stable. The best PQD-MAI device achieved an efficiency of 18.93% (certified at 18.06%), with 65 devices tested under each condition to validate the statistical results. The optimized devices also showed excellent stability, retaining 88% efficiency after 1200 h of continuous light exposure.

Most recently, several groups have reported PCEs approaching 20%. For example, Zhang et al. developed consecutive surface matrix engineering (CSME) with a PCE of 19.14% [207]. Zhao et al. introduced fluorinated pseudo-halide (PF_6_^−^) ligand shells that simultaneously passivate defects, enhance stability, and improve charge transport, reaching a PCE of 19.0% (17.2% for 1 cm^2^ devices) [208].

#### 5.4.2. Key Innovations in Perovskite Quantum Dot Solar Cells

##### Surface Chemistry and Ligand Engineering

The most crucial factor in the development of PQDSCs is ligand engineering. Simple ligand displacement with small organic compounds, such as formamidinium iodide, was employed in early devices. However, this method often led to additional defect states, uneven surface coverage, and inadequate ligand removal.

The development of Surface Matrix Curing (SMC) and Solvent Mediated Ligand Exchange (SMLE) protocols represented a fundamental shift from simple ligand displacement to comprehensive surface reconstruction [209,210]. These methods acknowledge that to achieve successful QD coupling, the inorganic matrix, which provides mechanical stability and electrical connectivity, must be actively rebuilt while removing insulating ligands. Key innovations include: (1) controlled ligand removal, using mild polar solvents that selectively remove excess ligands without damaging QD cores. (2) Surface matrix reconstruction: employing short, bifunctional ligands that can simultaneously passivate surface defects and promote interdot coupling. (3) Systematic optimization: developing processing protocols that systematically optimize ligand density, exchange completeness, and film morphology

Recent advances have introduced even more sophisticated approaches, including dual-ionic ligand systems [211] and fluorinated ligand architectures [208] that provide enhanced passivation while maintaining excellent charge transport properties.

##### Compositional Engineering

Optimizing perovskite composition represents another crucial innovation pathway. Early devices focused primarily on CsPbI_3_ due to its good phase stability, but this composition suffers from a suboptimal bandgap (~1.73 eV) for single-junction photovoltaics. The introduction of mixed-cation Cs_1−x_FA_x_PbI_3_ systems provided a pathway to simultaneously optimize bandgap and stability [204].

Mixed-cation strategies offer several advantages: (1) Bandgap tuning: systematic adjustment of the Cs:FA ratio enables precise bandgap control from ~1.73 eV (CsPbI_3_) to ~1.48 eV (FAPbI_3_). (2) Enhanced stability: the entropic stabilization effect of cation mixing improves the phase [212] stability compared to single-cation systems. (3) Processing tolerance: mixed-cation QDs show greater tolerance to ligand exchange conditions, enabling more aggressive surface treatments

Recent work has also explored B-site engineering through Pb-Sn alloying, though oxidation stability remains a significant challenge for tin-containing systems.

## 6. Perspective

Future work on QDSCs will focus on at least some of the following directions: (I) QD active-layer materials, (II) material interfaces, and (III) device architecture.

### 6.1. Active-Layer Materials

As we have reviewed so far, PQDs appear to be the most effective active layer for QDSCs, achieving high PCEs. Unfortunately, device durability/stability is the key issue for perovskite-based SCs. Therefore, there is room for other QD materials to supplement perovskite QDs. Besides CQDs, researchers have started to develop several other environmentally compatible QDs, such as MoS_2_ QDs [213,214] and BN QDs [214,215]. It should be noted that BN QDs are not actually QDs of BN. The fluorescence is due to defect and impurity states in the nanoparticles [216]. Since MoS_2_ QDs are synthesized by reflux methods similar to BN QDs, the contribution of defect and impurity states to the detected fluorescence properties is worth further investigation.

### 6.2. Materials Interfaces

Continued enhancement in PCEs appears to require more than improvements in the interfaces of active layers (QDs). Researchers remain vigilant in seeking to overcome limitations at the materials interfaces between QD-ETLs and QD-HTLs, for example. Our recent work suggested that high-quality ETLs and HTLs could reduce charge-trapping states near the interfaces. A PCE of 11.4% was obtained from our ITO/ZnO/core–shell CdSe/ZnS QDs/MoO_3_/gold devices [174], with *V_oc_* approaching the theoretical limit of ~2 V for ZnO-based devices.

### 6.3. Device Architecture

The most important aspect of future research should focus on circumventing the Shockley–Queisser limit by incorporating multiple material junctions to form a solar cell module. We will narrow our scope on a few trends in advanced QDSC device designs: (a) inverted vs. conventional stacking, (b) bandgap-graded single-junctions or multiple/tandem junctions, and (c) hybrid module layouts.

#### 6.3.1. Inverted vs. Conventional Stacking

Conventional QDSCs are generally stacked in the ITO/ETL/QDs/HTL/metal layered structure. In this configuration, the junction is between the QDs and the top layer, resulting in the depletion region at the far end of the illuminated side at the ITO end. In an inverted structure, the ETL and HTL locations are effectively swapped, placing the depletion region on the light-illuminated side, which could, in turn, increase photon harvesting in short-wavelength regions. In their 2018 work, Wang et al. fabricated an inverted device using iodine-capped n-type PbS QDs with a p-type NiO HTL to achieve heightened carrier extraction and transport, over-doubling the PCE from 4.2% to 9.7% [217].

#### 6.3.2. Bandgap-Graded Single-Junctions or Multiple/Tandem Junctions

An advantage of QDs is their bandgap tunability. By controlling the particle size or composition, the bandgap of a colloidal QD solution can be tuned. Advanced QDSC architecture utilizes this feature to create bandgap-graded active layers in which QDs of different sizes (and thus band gaps) are spatially distributed within the device. This spatial separation of QDs with different band gaps creates built-in energy gradients that broaden the wavelength range of absorbed light, while also facilitating directional carrier transport and suppressing recombination. In such a configuration, the wide-bandgap (smaller-sized) QDs are placed near one electrode to block undesired carrier leakage or recombination, while lower bandgap (larger-sized) QDs are placed near the opposite electrode to absorb longer wavelength photons. Recent studies have shown that the average carrier lifetime in graded devices can be over twice that in ungraded devices [218]. One of the early examples of this was reported by Wang et al. in their 2011 study, which used multiple sizes of PbS QDs to fabricate proof-of-concept devices with PCEs around 4% [219]. These graded devices operate as pseudo-tandem cells, achieving enhanced performance while maintaining the simple single-junction design. Future work can further enhance efficiency by improving surface passivation and optimizing transport layers.

#### 6.3.3. Hybrid Module Layouts

Multijunction devices could likely overcome the Shockley–Queisser limit. This method can be readily extended to QD-based solar technology—the high tunability of QD band gaps across a wide spectrum makes them especially well-suited for tandem devices. There are examples in the literature using both all-QD stacks [101] or hybrid stacks, consisting of QDs along with other materials, such as perovskites or organics [220]. Perovskite/QD tandem cells have been fabricated in both two-terminal and four-terminal configurations. In one study, a two-terminal perovskite/PbS QD tandem cell achieved a ~17% PCE, while a four-terminal device achieved ~21% under ambient conditions. Notably, the devices exhibited operational stability under continuous illumination, losing only 6% PCE over 500 h of illumination [221]. Additionally, the devices maintained their performance, with only minor changes, after 70 days of exposure to 65% relative humidity.

While future research focuses on these three directions, we must also study how to produce fourth-generation SCs that are more durable, scalable, and cost-effective than the single-crystalline Si SCs that we defined in Section 3.3. These SCs must also be compatible with humans and the environment and add new functionality, such as being flexible and wearable, without being limited by any form factors.

To sum up, this article provides a comprehensive review of the theory of photovoltaic devices, materials (QDs, ETLs, and HTLs), and device architectures. The future perspective outlined here may pave the way towards more efficient and reliable QDSCs as the fourth-generation architecture.

## Figures and Tables

**Table 2:** Common HTL materials in QDSCs.

HTL Material	Type	Key Properties	Advantages	Limitations	References
**PEDOT:PSS**	Organic polymer	High conductivity, solution processable	Smooth film formation, easy processing	Moisture sensitive	[155,161,169]
**Spiro-MeOTAD**	Organic	High hole mobility	High device efficiency	Dopant instability	[170,171]
**MoO_3_**	Inorganic	High work function	Excellent valence band alignment	Vacuum deposition is often required	[145,154,157]
**NiO**	Inorganic	Wide bandgap p-type oxide	High stability, suitable for p–i–n devices	Lower conductivity	[158,164]
**CuO/CuI/CuSCN**	Inorganic	High hole mobility	Improved stability compared with organics	Processing complexity	[159,164]

## Data Availability

No new data were created or analyzed in this study.
